# Task complexity and location specific changes of cortical thickness in executive and salience networks after working memory training

**DOI:** 10.1016/j.neuroimage.2016.01.007

**Published:** 2016-04-15

**Authors:** Claudia Metzler-Baddeley, Karen Caeyenberghs, Sonya Foley, Derek K. Jones

**Affiliations:** a Cardiff University Brain Research Imaging Centre (CUBRIC), School of Psychology, and Neuroscience and Mental Health Research Institute (NMHRI), Cardiff University, Cardiff CF10 3AT, UK; b School of Psychology, Australian Catholic University, Melbourne, Australia

**Keywords:** Working memory training, Cortical thickness, Executive network, Salience network, Insula, Parieto-frontal regions, Activity-dependent brain plasticity

## Abstract

Novel activities and experiences shape the brain's structure and organisation and, hence, our behaviour. However, evidence from structural plasticity studies remains mixed and the neural correlates of learning and practice are still poorly understood. We conducted a robustly designed study into grey matter plasticity following 2 months of working memory training. We generated a priori hypotheses regarding the location of plastic effects across three cognitive control networks (executive, anterior salience and basal ganglia networks), and compared the effects of adaptive training (*n* = 20) with a well-matched active control group (*n* = 20) which differed in training complexity and included extensive cognitive assessment before and after the training. Adaptive training relative to control activities resulted in a complex pattern of subtle and localised structural changes: Training was associated with *increases* in cortical thickness in right-lateralised executive regions, notably the right caudal middle frontal cortex, as well as *increases* in the volume of the left pallidum. In addition the training group showed *reductions* of thickness in the right insula, which were correlated with training-induced improvements in backward digit span performance. Unexpectedly, control activities were associated with reductions in thickness in the right *pars triangularis*. These results suggest that the direction of activity-induced plastic changes depend on the level of training complexity as well as brain location. These observations are consistent with the view that the brain responds dynamically to environmental demands by focusing resources on task relevant networks and eliminating irrelevant processing for the purpose of energy reduction.

## Highlights

• Training led to *increases* of cortical thickness in right parieto-frontal cortex.
• Training led to a *reduction* of thickness in the right insula.
• Changes in insula were related to changes in working memory span.
• Control activities led to thickness *reductions* in right *pars triangularis*.
• Patterns of brain plasticity could correctly classify 80% of trainees.

## Introduction

The human brain is known to respond adaptively to varying environmental demands (Pascual-Leone et al., 2005). There is general consensus that in addition to our genes, novel activities and experiences may shape the brain's structure and organisation and, hence, our behaviour. However, the neural correlates of learning in the human brain still remain poorly understood. Magnetic resonance imaging (MRI) techniques provide non-invasive tools to investigate training induced brain plasticity *in vivo* and hence may aid our understanding of the neural substrates underpinning learning and practice effects.

Evidence for training-induced brain plasticity remains inconsistent and controversial (Thomas and Baker, 2013). Longitudinal studies into macrostructural brain plasticity have found mixed findings regarding the direction of training-induced alterations in grey matter volume or cortical thickness in task-relevant brain regions (Draganski and May, 2008, Engvig et al., 2010, Mårtensson et al., 2012, Takeuchi et al., 2011, Taubert et al., 2010) (see for review Thomas and Baker, 2013, Valkanova et al., 2013). For instance Draganski et al. (2004) found *increases* in grey matter volume in temporal and parietal regions after 3 months of juggling whilst Takeuchi et al. (2011) reported *reductions* of grey matter volume in parieto-frontal cortical regions after working memory training.

A number of factors may explain these inconsistencies and point to a need for more empirical evidence based on well-controlled studies using a rigorous experimental design (see Thomas and Baker, 2013, Valkanova et al., 2013). For instance, experimental control of the nature of activities undertaken by the comparison groups in previous studies was often weak or not present. Previous studies also varied considerably in terms of activity and length of training interventions as well as the chosen behavioural and imaging outcome measures. Further, the neural mechanisms thought to contribute to the net changes in MRI indices include complex processes of neurogenesis, synaptogenesis, glia cell and myelin formation and selective pruning of nerve fibres (Zatorre et al., 2012). These mechanisms are likely to operate in a dynamic fashion and on different time scales and hence may lead to different effects depending on the precise training conditions and the brain region in which observations are made (Pajevic et al., 2014, Zatorre et al., 2012).

The present study followed recent recommendations (Thomas and Baker, 2013) and addressed many of the problems above to shed further light on the nature of training-induced cortical plasticity. An established working memory training (Cogmed, 2012) targeting the ability to maintain and manipulate information temporarily (Baddeley, 1996) was chosen because of its demonstrated efficacy in terms of improving working memory span capacity (Holmes et al., 2009, Klingberg, 2010).

Participants were asked to train their working memory using computerised verbal and spatial working memory span tasks for 2 months (Cogmed, 2012). To ensure that participants trained at an optimally challenging level, task difficulty was increased or decreased adaptively depending on a trainee's performance levels (adaptive training). Importantly, the effects of adaptive training on cognition and brain structure were compared to an active control condition in which the same working memory tasks were performed for the same number of sessions (40 in total) but at a constant low level of difficulty (non-adaptive control). Thus the two groups were exposed to the same number and order of learning activities in the same computer environment but differed in terms of the level of training complexity.

Working memory training was also chosen because of evidence from neuroimaging studies of localised structural and functional alterations in parieto-frontal regions: Reduced grey matter volume in dorsolateral prefrontal and parietal cortices (Takeuchi et al., 2011), increased diffusion MRI derived fractional anisotropy around the intraparietal sulcus (Takeuchi et al., 2010), increased functional MRI activity in right middle frontal gyrus, inferior parietal and intraparietal cortices (Olesen et al., 2004) and changes in D1 receptor binding potential in right dorsal and ventral frontal and posterior cortices (McNab et al., 2009).

In addition there is a large body of evidence based on imaging and lesion studies that parieto-frontal regions, notably the dorsolateral prefrontal cortex and posterior parietal cortex, form part of a central executive network important for the control of goal-directed actions in working memory (Duncan and Owen, 2000, Owen et al., 1990). The executive network is thought to interact with anterior salience and basal ganglia regions to support efficient working memory processes (Dosenbach et al., 2008, Dosenbach et al., 2006, Menon and Uddin, 2010). Anterior insula and anterior cingulate cortex detect salient stimuli and initiate executive control (Menon and Uddin, 2010) whilst the basal ganglia regulate the information flow into working memory and the selection of goal-directed responses via inhibitory feedback loops (Gurney et al., 2001a, Gurney et al., 2001b, McNab and Klingberg, 2008, McNab et al., 2008, Redgrave et al., 2010, Redgrave et al., 2011). Temporal dynamic changes in the striatum have also been observed after working memory-updating training (7 versus 50 days) suggesting a potential role of the basal ganglia in mediating working memory function (Kühn et al., 2013).

**Fig. 1:**
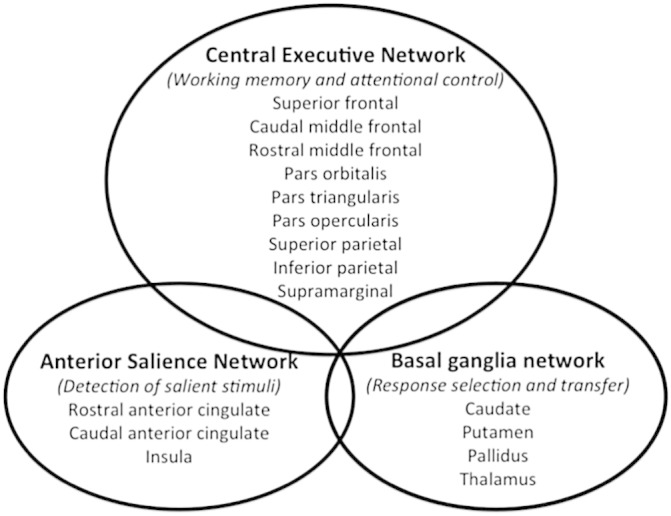
Schematic representation of cortical and sub-cortical regions of cognitive control networks and their proposed function. Target regions of the central executive, anterior salience and basal ganglia networks were selected from the Desikan–Killiany atlas (Desikan et al., 2006). Executive parieto-frontal regions selected were the inferior parietal cortex, superior parietal cortex, supramarginal gyrus encompassing intraparietal sulcus, caudal middle frontal gyrus, rostral middle frontal gyrus, superior frontal cortex as well as *pars opercularis*, *pars triangularis*, and *pars orbitalis* of the inferior ventro-lateral prefrontal cortex. Anterior salience network regions selected were the insula and caudal and rostral anterior cingulate cortices. Sub-cortical regions of the basal ganglia, i.e., caudate, putamen, and *globus pallidum* and the thalamus were also included in the analyses. All regions were included from both hemispheres.

Thus based on ample evidence regarding brain regions involved in working memory functions combined with the results from previous working memory plasticity studies, it was possible to formulate hypothesis-driven predictions regarding the localisation of the expected structural alterations, hence yielding anatomical specificity (Thomas and Baker, 2013). We utilised the known anatomy of the central executive, anterior salience, and basal ganglia networks as well as the results by Olesen et al. (2004) to guide the selection of cortical and subcortical regions of interest (ROI) from the Desikan–Killiany parcellation scheme (2006) (see Fig. 1).

An ROI approach was chosen because it is already known that training may lead to localised changes in brain networks that support the trained activity and that such plastic changes are usually small in effect size (Thomas and Baker, 2013, Valkanova et al., 2013). For instance, memory training in older people was found previously to increase cortical thickness by around 0.05 mm (Engvig et al., 2010). Thus the rationale for focusing on the selected ROIs was to increase the power of our study to detect localised and subtle training effects across different cognitive control networks.

For each ROI we measured average cortical thickness or subcortical volume respectively at baseline and outcome time points. Cortical thickness measures were derived from the FreeSurfer longitudinal analysis pipeline (Reuter et al., 2012) which was chosen due to the demonstrated precision (Lerch and Evans, 2005), reproducibility (Reuter et al., 2012), sensitivity to longitudinal changes (Engvig et al., 2010, Sabuncu et al., 2011) and consistency with post mortem data (Fischl and Dale, 2000).

We hypothesised that 2 months of adaptive working memory training would lead to alterations in cortical thickness in parieto-frontal executive network regions notably in the middle frontal gyrus, inferior parietal and intra-parietal cortices of the right hemisphere (e.g. Olesen et al., 2004). Although previous studies provided guidance with regard to which brain regions were expected to alter their thickness, it was more difficult to generate hypotheses regarding the direction of change. Based on findings of increased receptor density we expected to observe an increase in cortical thickness in parieto-frontal regions likely due to an increase in neural activity and hence an increase in synapses, receptors and myelin-forming glia cells. However, Takeuchi et al. (2011) reported reductions of grey matter volume in parieto-frontal regions suggesting that training effects are more complex and may depend on the precise training schedule and location in the brain.

Similarly, working memory training has been associated both with a decrease of fMRI BOLD activity (Olesen et al., 2004) and an increase in functional resting state connectivity of the anterior cingulate, a region of the anterior salience network (Jolles et al., 2013). Although structural and functional MRI measures are not directly comparable, we utilised these findings to hypothesise that adaptive training would result in changes in anterior salience network regions, i.e., in the anterior cingulate and the insula; however, the direction of plastic changes to be expected remained unclear (Jolles et al., 2013, Olesen et al., 2004).

Participants were tested before and after the training in a number of near- and far-transfer working memory and executive function benchmark tasks (Owen et al., 2010). We assumed that training-induced plastic alterations would be relevant for working memory functions and hence hypothesised any potential structural changes to correlate with training specific cognitive improvements. Finally, the question of whether reasoning and executive function abilities can be improved by working memory training remains controversial (Melby-Lervåg and Hulme, 2013, Shipstead et al., 2012, von Bastian and Oberauer, 2014). Recent meta-analyses on transfer effects of *n*-back working memory training concluded that positive albeit small effects may be detected (Au et al., 2015). However these results have been questioned by Melby-Lervag and Hulme (2015) who did not find evidence for transfer effects of working memory training on non-verbal reasoning after restricting their meta-analyses to more rigorous studies that included active control groups and after taking baseline differences between groups into account in their effect size calculation. In the present study we expected any potential transfer effects to be associated with changes in the structure of the caudate and putamen based on previous reports of generalisation effects being mediated by the striatum of the basal ganglia (Dahlin et al., 2008).

## Materials and methods

This study was approved by the Cardiff University School of Psychology Ethical Committee and all volunteers provided informed written consent.

### Participants

**Fig. 2:**
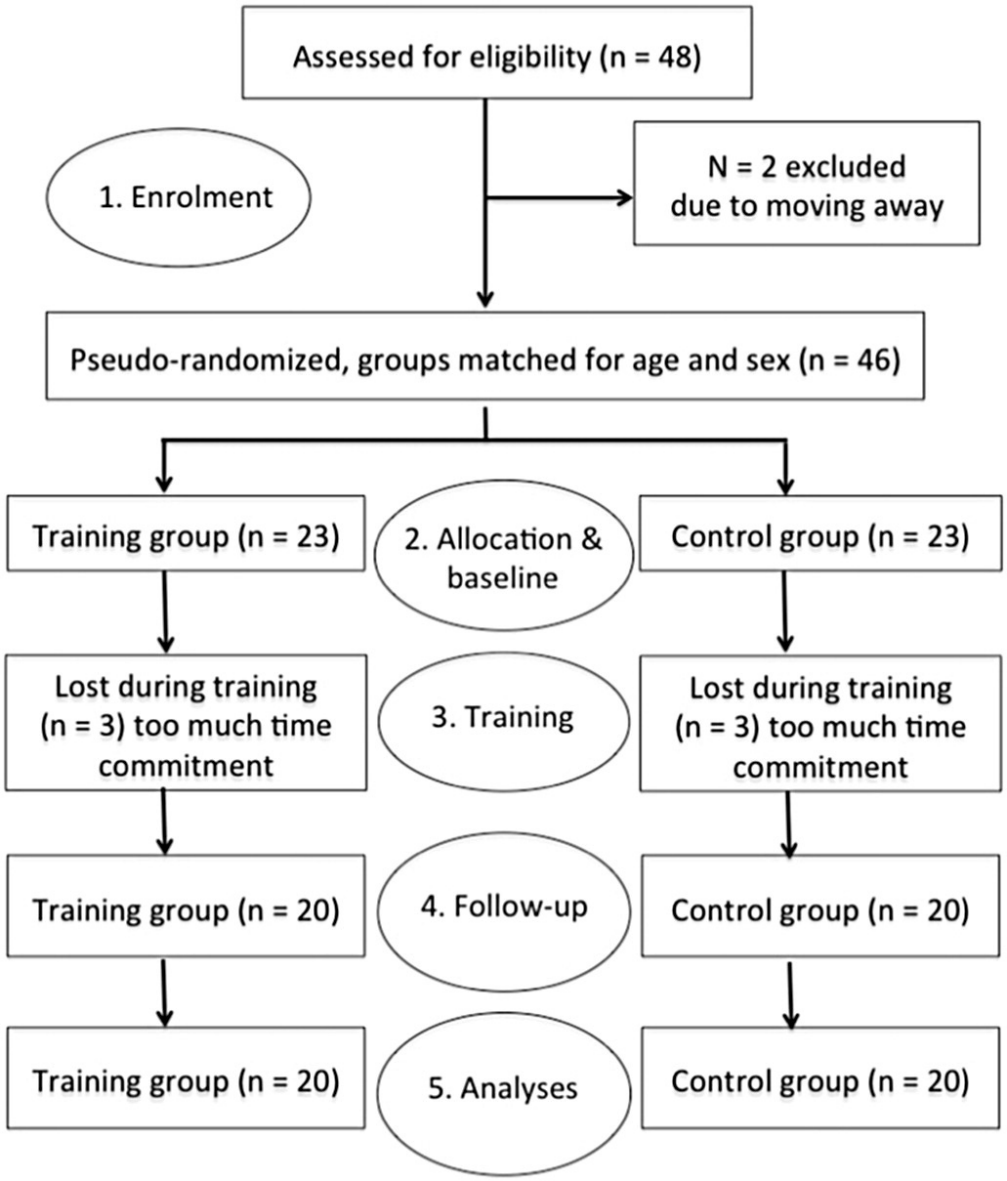
Flow diagram following the CONSORT guidelines (http://www.consort-statement.org/consort-statement/flow-diagram) of participants' progress through the different study stages of enrolment, training allocation, baseline MRI and cognitive assessment, working memory training phase, follow-up MRI and cognitive assessment and data analyses.

Fig. 2 gives an overview over the different stages of the study and the number of participants in each stage following the CONSORT guidelines for reporting randomised controlled trials (http://www.consort-statement.org/consort-statement/flow-diagram). Forty-eight eligible volunteers (19–40 years of age) were recruited from the Cardiff University volunteer databases and from the local community. Participants were eligible to take part in the study if they had normal or corrected vision and no history of neurological or psychiatric illness, drug/alcohol abuse or MRI contraindications. They also were required to have a good command of English and access to a computer and internet connection for home training. Two recruited participants did not commence the training after the baseline assessment because they moved away. The remaining 46 participants were pseudo-randomly allocated to either the training or the control group with the provision that the groups were matched for gender and age. All participants were blind to their training condition.

Six individuals, three from the training and three from the control group withdrew during the study due to the significant time commitment of the training. Forty participants, *n* = 20 individuals in the adaptive training and *n* = 20 in the low capacity control group were compliant with and completed the training and all behavioural and MRI outcome assessments. The data of these 40 participants were included in the final analyses. Only one participant in the training group was left-handed, all other participants were right-handed. Besides age and sex, the groups were also matched for baseline cognitive performance in a number of working memory and executive function computerised benchmark tests (Table 1) known to correlate with general intelligence (Hampshire et al., 2012) (please note though that the training group showed a non-significant trend for better performance in the self-ordered search task). Thus, both groups were comparable in terms of demographic variables and intellectual abilities (Table 1). All participants completed 40 training sessions with the same number of span tasks per session (*n* = 7) presented in the same order. The control group, however, practiced at a shorter span length of 3 items per trial and as a consequence exercised on average 7 min less than the training group (Table 1).

### Working memory training

**Fig. 3:**
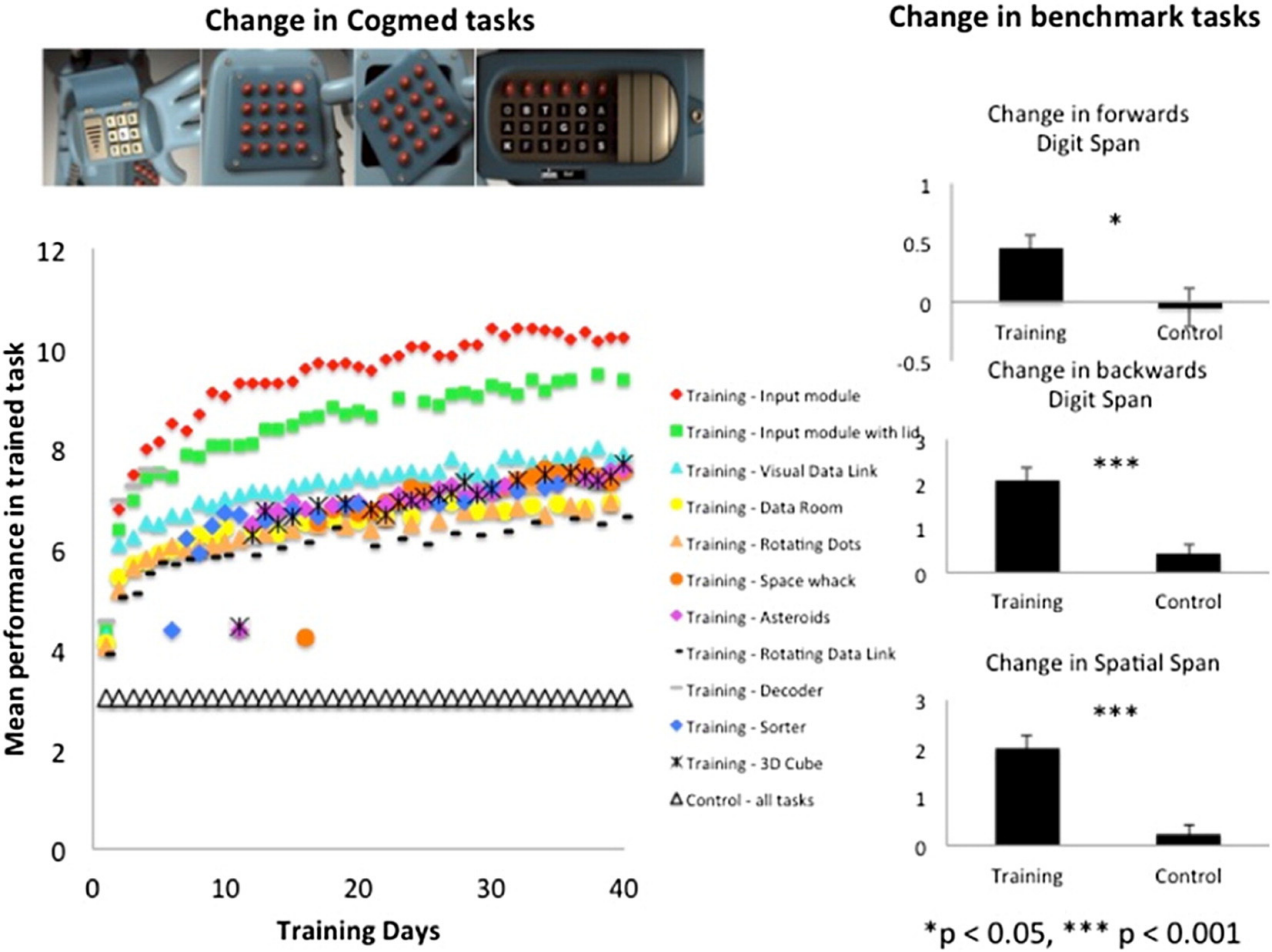
Left hand panel: Average performance scores in the trained working memory span tasks (Cogmed, 2012) across 40 training sessions. Performance of the *adaptive training* group in the eleven tasks is plotted with coloured symbols. Performance of the *non-adaptive control* group is plotted with white triangles at the bottom of the graph. Control performance remained flat at a level of three item spans in all tasks throughout the training. Some examples of digit and spatial span displays can be seen at the top of the figure. Right hand panel: Adaptive working memory training relative to the control condition improved performance in the working memory span benchmark tasks of backward digit span and spatial span. A trend for improvement was observed for forward digit span. *p ≤ 0.05, ***p ≤ 0.001.

Working memory capacity was trained with computerised exercises of verbal (e.g. digits) and spatial (e.g. flashing lights) span tasks under various conditions such as repeating sequences of auditory digits in forward or backward order, with and without visual cues, repeating sequences of flashing lights in stationary or rotating displays or repeating sequences of associated verbal and spatial information (Cogmed, 2012).^1^ Examples of some of these exercises are depicted in top left hand panel of Fig. 3.

Participants were provided with a training booklet that included instructions for the training schedule as well as optimal training conditions such as making sure to exercise in an environment free of distractions. They were provided with their individual training account and accessed their training via the internet from home. They were asked to practice five times per week for eight weeks i.e., to complete 40 training sessions in total.

In each training session the participants had to complete seven out of eleven possible training tasks. Therefore not all eleven training exercises were introduced at the first training session but five of them were first encountered at later stages (as indicated by the isolated symbols in Fig. 3 left panel graph). Training tasks, frequency and order of tasks were identical for all participants. All training trials for each participant were recorded automatically for each task and training session so that compliance with the training and training progress could be monitored on a daily basis.

All participants were provided with feedback regarding their compliance and training progress once per week by email. For all participants and all exercises, the training started at a level of two items per span. All participants were blind to the training condition they were allocated to.

*Adaptive training*: Task difficulty increased or decreased adaptively depending on the trainee's level of performance. If a participant repeated a sequence of *n* items successfully, the same number of items was presented twice further in more challenging displays until three trials of a given span length were repeated correctly and he/she could proceed to *n* + 1 items. The same procedure was applied when participants made mistakes and the level of difficulty was reduced to *n* − 1 items.

*Non-adaptive control training*: The participants trained on a maximum level of three item spans independently of their performance throughout all training days. Since training sessions expanded in length with increasing training complexity for the adaptive training group, the length of training in terms of the number of repetitions was also increased each week for each individual in the control group to match average training times for both groups as closely as possible.

### Cognitive benchmark assessment

The participants were assessed before and after the training, on a number of computerised working memory and executive function tasks from the Cambridge Brain Sciences Laboratory (www.cambridgebrainsciences.com) (Owen et al., 2010) as well as the automated symmetry span task (Unsworth et al., 2005). Outcome assessments of cognitive functions were performed for each participant within a week of completing the final training session.

Verbal and spatial working memory spans were assessed with computerised versions of the *forward and backward digit span* and the *spatial span tasks* (Wechsler, 1999). Task difficulty was adjusted by increasing the number of span items by one following a successful trial and decreasing by one following an unsuccessful trial. Outcome measures were the average number of digits or spatial locations respectively in all successfully completed trials.

The ability to maintain and manipulate spatial information was assessed with the *self-ordered spatial span task* (Owen et al., 1990). In this task a token is hidden in one of several boxes on the computer screen. The participants' task was to find the token by clicking on the boxes and to remember the location of the token since novel tokens were never hidden in previously occupied boxes. The participants completed a trial successfully when all targets had been found with the outcome measures being the average number of successfully completed trials.

The ability to suppress distracting and response conflicting information was assessed with the “*double trouble*” *task*, a version of the Stroop task (Stroop, 1935). The participants saw a target colour word and two response colour words on the screen and had to select the word that correctly described the target ink colour. Task difficulty was manipulated by varying the congruency between the ink colour and colour meaning of the target and response words. The outcome measure was the number of correct responses within 90 s.

Complex verbal reasoning was assessed with an adapted version of the *grammatical reasoning* test (Baddeley, 1968), which required participants to decide whether grammatical statements about a presented picture such as “the green circle is within the red big triangle” were correct or false, answering as many questions as quickly as possible within 90 s. The outcome measure was the total number of trials answered correctly minus the number answered incorrectly.

Nonverbal abstract reasoning was assessed with the *odd one out* task, an adaptation of the Raven's Progressive Matrices (Raven, 1936), in which participants were presented with nine patterns on the screen, each made up of colour, shape, and number features and had to find the one pattern that differed from the others either according to a single feature or a combination of features. The outcome measure was the number of trials correctly solved within 3 min.

The ability to plan and think forward was assessed with the *Hampshire tree task*, a version of the Tower of London/Hanoi test (Shallice, 1982, Simon, 1975). Participants were presented with a tree-shaped frame on the screen with nine numbered balls slotted onto the branches and had to rearrange the balls so that they were ordered numerically with as few moves as possible. Participants could only move one ball at a time and only balls that were not blocked by another ball. The time limit for this task was 3 min and the outcome measure was the number of correctly executed moves with fewer moves reflecting better performance.

Finally, the ability to multi-task was assessed with the *automated symmetry span* task (Unsworth et al., 2005), which requires participants to rapidly alternate between repeating spatial spans of increasing length and symmetry judgments for patterns of increasing complexity. The outcome measure used in this study was the total number of correctly completed trials.

### MRI data acquisition

MRI data were acquired on a 3 T General Electric HDx MRI system (GE Medical Systems, Milwaukee, USA) using an eight channel receive-only head RF coil at the Cardiff University Brain Research Imaging Centre (CUBRIC). MRI sessions were interleaved for both groups, i.e., it was ensured that participants from one group were not block-scanned to avoid confound between the experimental conditions and scanner-related changes in data acquisition such as scanner drift effects or time-of-scanning effects (Nakamura et al., 2015). The two groups were comparable in terms of the average time of day for baseline and outcome scanning sessions (Table 1). Time of scanning also did not differ between baseline and outcome sessions for each group [Training: *t*(19) = 0.66, *p* = 0.52; Controls: *t*(19) = − 0.96, *p* = 0.35]. For all participants MRI outcome assessments took place within a week of completion of the last training session. High-resolution T_1_-weighted anatomical images were acquired (FSPGR) (256 × 256 matrix, TR = 7.8 ms, TE = 2.9 ms, flip angle = 20, 172 slices, 1 mm slice thickness, FOV = 23 cm, 7 min acquisition time). All images were inspected visually in MRIcron (Riordan et al., 2007) for motion-related artefacts such as blurring, ghosting and striping, as well as general criteria than can affect image quality, e.g. head coverage, ringing artefacts, or signal inhomogeneity.

### FreeSurfer analyses

The longitudinal stream of FreeSurfer version 5.3 (http://surfer.nmr.mgh.harvard.edu) was used to improve the robustness and sensitivity of the longitudinal analyses of the cortical thickness of brain regions of using a semi-automated approach described in detail elsewhere (Fischl, 2012, Jovicich et al., 2009), with the use of additional computing resources from the high performance computing TIER1 cluster at the University of Gent (http://www.ugent.be/hpc/).

The longitudinal scheme is designed to be unbiased with respect to any specific time point (Thomas and Baker, 2013), by creating a within-subject template space and image. For this stream, the T_1_-weighted scans of the two time points were processed cross-sectionally. This processing included removal of non-brain tissue using a hybrid watershed/surface deformation procedure, automated Talairach transformation, segmentation of the subcortical white matter and deep grey matter volumetric structures (including hippocampus, amygdala, caudate, putamen, ventricles) (Fischl et al., 2002, Fischl et al., 2004) intensity normalisation, tessellation of the grey matter white matter boundary, automated topology correction (Fischl et al., 2001, Ségonne et al., 2004), and surface deformation following intensity gradients to optimally place the grey/white and grey/cerebrospinal fluid borders at the location where the greatest shift in intensity defines the transition to the other tissue class (Dale and Sereno, 1993, Fischl and Dale, 2000, Fischl et al., 1999). The results for each subject at each time point were carefully inspected to ensure the accuracy of the skull stripping, segmentation and cortical surface reconstruction. Where needed, appropriate manual corrections were performed as per the FreeSurfer tutorial (http://surfer.nmr.mgh.harvard.edu/fswiki/FsTutorial) either by (i) adding control points to help FreeSurfer identify the WM voxels (3 cases); (ii) replacing parts of the brain which were wrongly removed (5 cases); (iii) removing the skull and dura in case they were considered as parts of the brain (7 cases). Furthermore, an unbiased within-subject template space and image was created using robust, inverse consistent registration (Reuter et al., 2012).

The processing steps as described above were then initialised with common information from the within-subject template, resulting in increased reliability and statistical power. Finally, automated cortical parcellation and region of interest definition was performed using the Desikan–Killiany atlas (Desikan et al., 2006) resulting in mean cortical thickness estimations calculated from all vertices within the selected twelve cortical parcellations per hemisphere as well as sub-cortical volume measures for the basal ganglia and the thalamus. We selected cortical thickness in our main statistical analyses, because cortical thickness correlates with quantitative histological measures of neuronal density (Rosas et al., 2002). Moreover, the cortical thickness maps produced are not restricted to the voxel resolution of the original data, allowing us to detect sub-millimeter differences between groups and time points.

### Statistical analyses

Statistical analyses were carried out in SPSS Version 20.0 (IBM, 2011). All data were inspected for outliers defined as values more than two times the standard deviation from the average cognitive or structural measure. Outliers were defined separately per time point, group and for difference scores.

#### Analyses of structural MRI data

##### Omnibus repeated measure ANOVAs of group × time × region-of-interest × hemisphere interaction effects

The demonstration of an interaction effect between group (training versus control) and time-point (pre-versus post-training) is generally seen as the gold standard evidence for training dependent changes (Thomas and Baker, 2013). Thus we employed repeated measure analyses of variance (ANOVA) to investigate the effects of group (adaptive versus control), time (pre versus post-training) and brain hemisphere (left and right) and region (the 16 brain regions of the executive, salience and basal ganglia networks summarised in Fig. 1) on cortical thickness and subcortical volume measures. A complex four-way interaction effect was followed up with additional two and three-way ANOVAs for each factor (group, time, hemisphere, region of interest) individually. To find out if changes in brain regions across time were observed for both groups, we tested for three-way interactions between time, region and hemisphere for the adaptive and the control groups separately. Since potential group differences in brain structure at baseline may impact on any intervention effects, we also assessed interaction effects between group and regions for the baseline and outcome data separately. To find out whether training had differential effects on regions of the left and the right hemispheres we tested for three-way interaction effects between group, time and region for each side of the brain separately. Finally, we explored time by group interaction effects for each region of interest. Each of these 32 interaction effects had to reach a significance level of *p* ≤ 0.0016 to comply with a familywise Bonferroni corrected significance level of *p* ≤ 0.05.

##### Principal component analyses of changes in each cognitive control network

To test our theoretical assumption that the selected ROIs would cluster into central executive, anterior salience, and basal ganglia networks, Pearson's correlation coefficients between cortical thickness and subcortical volume changes were calculated across the ROIs. Based on the results of the repeated measure ANOVAs, principal component analysis (PCA) was carried out for right-lateralised regions only. Recognising the fact that cortical thickness changes in ROIs within the same cognitive control network share variance, a summary index of “cortical change” was derived with PCA based on changes of cortical thickness and subcortical volume measures across all participants for regions of the central executive, the anterior salience and the basal ganglia networks separately. Here, a procedure was used with orthogonal rotation of the factor matrix, ensuring that the extracted components were independent from one another. The factor loadings reflect the strength of each variable in defining the factor with negative loadings indicating that a variable points to the opposite direction than the other variables. For the right-lateralised executive network a summary index of structural change was based on the first principal component derived from the changes in cortical thickness across the nine parieto-frontal regions summarised in Fig. 1. For the right anterior salience network the first principal component (PC) was based on changes in cortical thickness indices of the right anterior cingulate (caudal and rostral portions) and the right insula. For the right basal ganglia network the first PC was derived from changes in volume of the caudate, putamen, pallidus and thalamus. Individual PC projections or factor scores were then used as dependent variables in independent *t*-tests to investigate between group effects on structural changes across regions of the three networks on the right side of the brain. Bonferroni correction for multiple comparisons was applied and the result of each *t*-test had to reach *p* ≤ 0.02 to comply with an experimentwise corrected significance level of 0.05.

##### Logistic regression analysis to predict the type of intervention based on structural changes

To identify changes in brain structure that could separate the adaptive training from the control group, binomial logistic regression analysis with training category (adaptive versus control) as dependent variable and changes in average cortical thickness and sub-cortical volume as independent variables was conducted applying stepwise forward maximum likelihood estimation. The overall success rate of models that included structural predictor variables was assessed with *χ*^2^ goodness of fit statistics relative to models based on intercept only. The significance of individual logistic regression coefficients for each independent variable was tested with the Wald statistic. To avoid over-fitting of the data, a model was selected as final when all included predictors contributed significantly (*p* ≤ 0.05) and addition of predictors in subsequent steps resulted in a reduction of significant contributions from individual logistic regression coefficients. The so-derived model's ability to classify novel data correctly was further tested with 10-fold cross validation. The overall success rate of this cross-validation analysis was assessed with *χ*^2^ goodness of fit statistic to test significance from chance.

##### Within-group comparisons of structural changes across time

Those brain regions that were identified in the repeated measure ANOVAs and the logistic regression analyses as showing evidence for differential structural changes between the two groups were explored further to establish the direction of changes within each group. Repeated measure analyses between pre- and post-training cortical thickness measures were conducted for each group separately. The reliability of individual *pre-* versus *post*-training changes was assessed with the 95% confidence interval (CI) of change derived from bootstrap analyses based on 1000 samples (referred to as 95% CI in the results section). Cohen's effect size *d* (Cohen, 1988) was calculated with G*power Version 3.1.3 (Faul et al., 2009) and added to each within-group comparison.

#### Analyses of training effects on cognition

Changes in cognitive performance were calculated for the nine benchmark tests which are summarised in Table 1. Independent *t*-tests were conducted assessing between group effects on changes in cognitive performance across the nine benchmark tests. The results of each of the nine independent *t*-tests had to reach *p* ≤ 0.006 to comply with experimentwise Bonferroni corrected significance level of 0.05.

For the training group, performance in the eleven Cogmed tasks after the first versus the last training sessions were compared with paired *t*-tests to assess training-specific improvements. Note that this analysis could not be performed on the control data since participants performed at ceiling for all training tasks and sessions.

#### Correlations between changes in brain structure and changes in cognitive performance

To ascertain whether structural brain changes were related to training-induced performance improvements, Spearman's rho correlation coefficients were calculated between those cognitive measures that demonstrated training specific changes and those structural measures for which within-group comparison demonstrated a significant change. Correlations coefficients were obtained for the adaptive training and the control group separately and Fisher's *r* to *z*-transformation was used to compare transformed correlation coefficients *ρ*^′^ between the groups. Non-parametric correlation coefficients were chosen because correlations were calculated within each group and hence sample sizes were limited after exclusion of outliers. There were eight correlations per group in total, each of which had to reach *p* ≤ 0.006 to comply with experimentwise Bonferroni corrected significance level of 0.05.

#### Assessment of the test–retest reliability and accuracy of the FreeSurfer measurements and effects of group-differences in training duration

We assessed the test–retest reliability of the FreeSurfer-derived indices in the following ways: Firstly we calculated the intraclass correlation coefficient (ICC) of cortical thickness measures between the two time points (pre versus post-training) across all participants for the left occipital cortex, a control region that was expected to be unaffected by the training program. Secondly we compared intracranial volume measures (ICV) between the two time points for each group separately since these should not change with the intervention or across time. Finally we tested for scanner drift effects, which could have potentially affected thickness measures across time by plotting average cortical thickness measures from the occipital cortex for each acquired dataset against time.

Since the groups differed in overall training duration we also assessed with Pearson correlation coefficient whether duration of training was related to changes in brain structure and in cognitive performance across all participants.

## Results

### Training induced effects on brain structure

#### Results of omnibus repeated measure analyses to test for time x group interaction effects

Repeated measure ANOVA demonstrated a four-way interaction effect between group, time, hemisphere and brain region [*F*(15,570) = 2.84, *p* ≤ 0.001]. This complex interaction effect was followed up with additional ANOVAs for each factor (group, time, hemisphere, region) separately.

*Group*: The adaptive training group showed a significant interaction effect between time, region and hemisphere [*F*(15,285) = 4.56, *p* ≤ 0.001] which was not observed for the control group [*F*(15,285) = 0.33, *p* = 0.99].

*Time*: No three-way interaction effects were observed between group, region and hemisphere for the baseline data [*F*(15,570) = 1.39, *p* = 0.14] or the outcome data [*F*(15,570) = 1.4, *p* = 0.13] but group and region interacted significantly at both time points [baseline: *F*(15,570) = 2.239, *p* ≤ 0.005; outcome: *F*(15,570) = 2.035, *p* ≤ 0.012].

*Hemisphere*: An interaction effect between group, time and region was present for the right hemisphere [*F*(15,570) = 1.87, *p* ≤ 0.024] but not for the left hemisphere [*F*(15,570) = 0.15, *p* = 1.0].

**Fig. 4:**
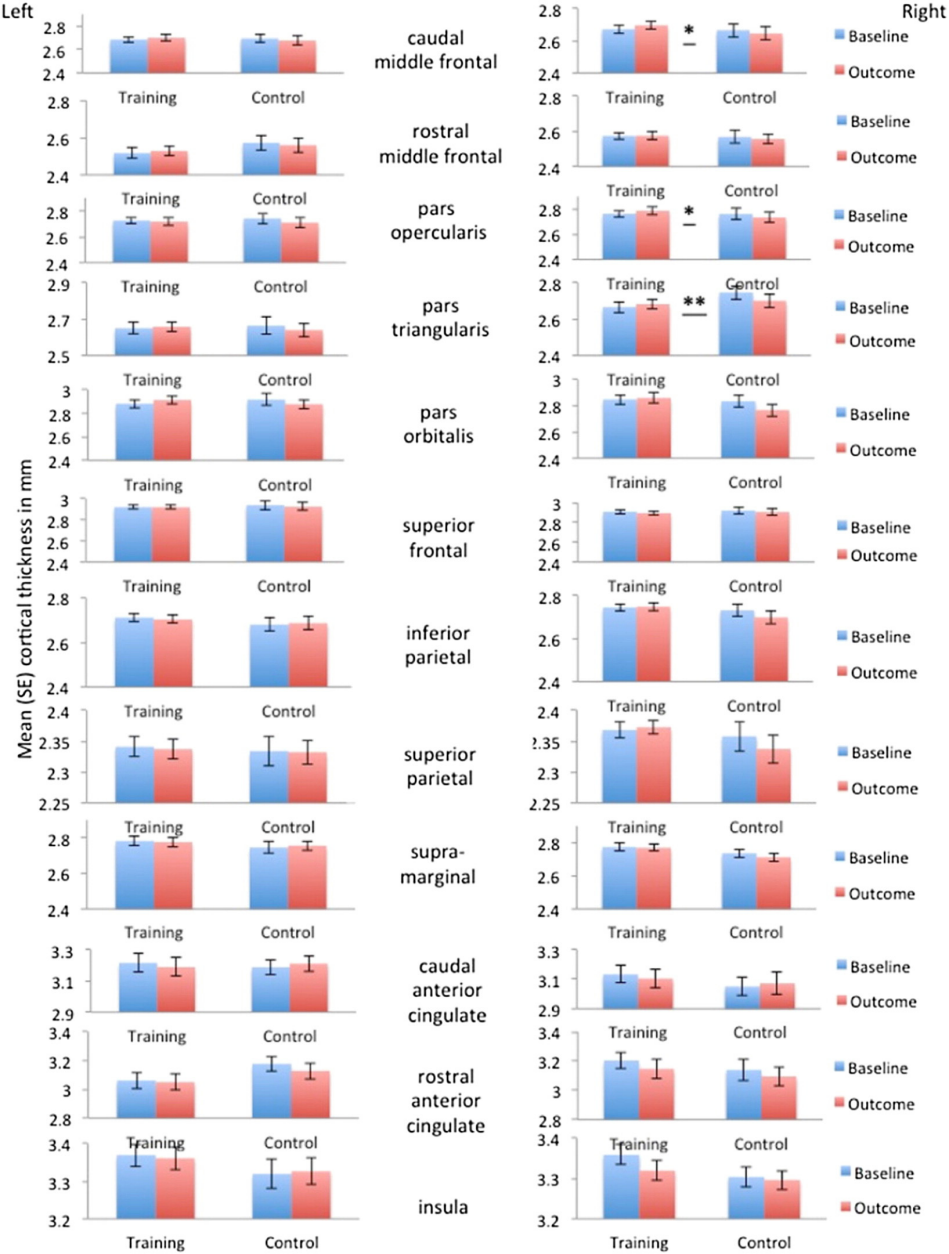

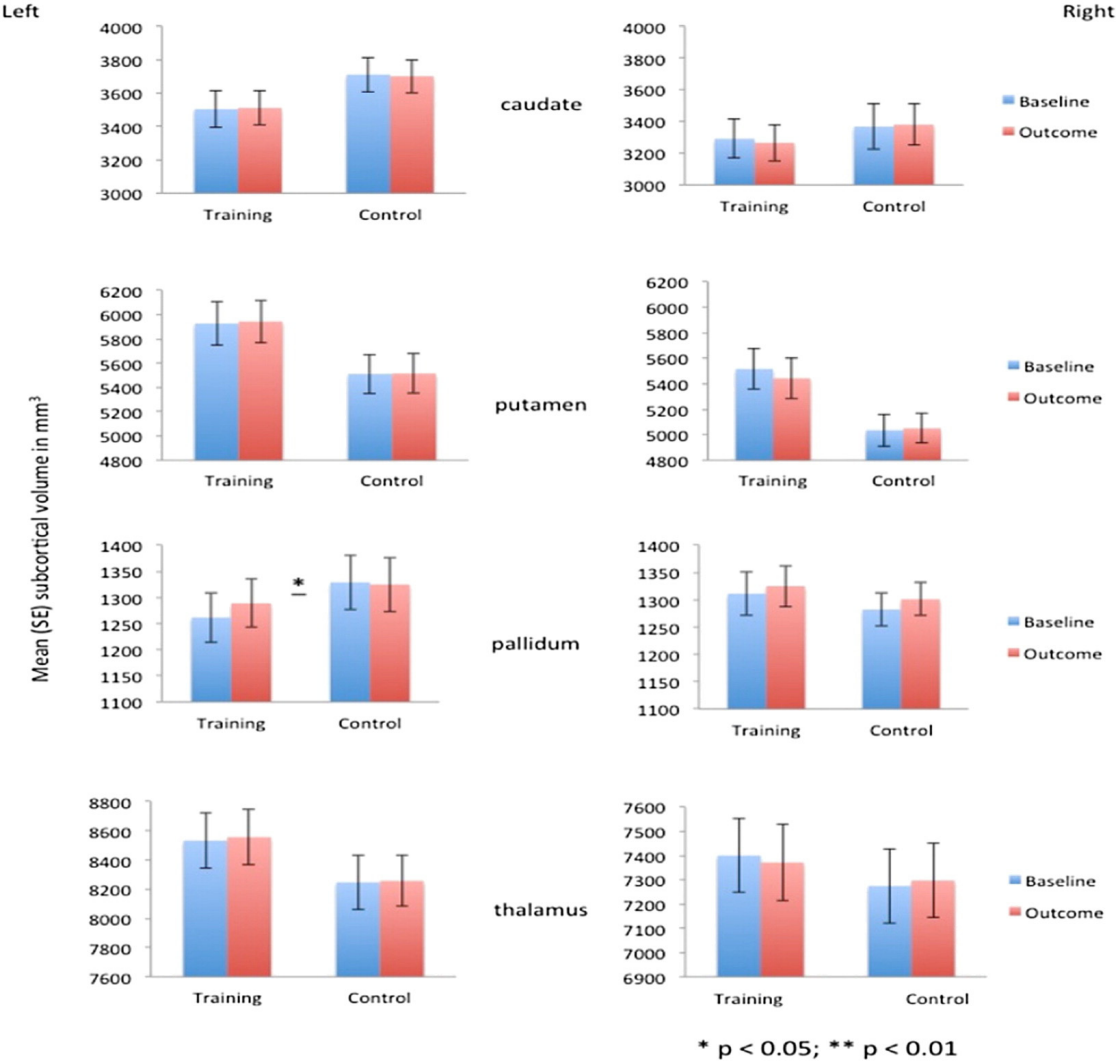
Bar graphs of the average cortical thickness/subcortical volume measures (standard error) as a function of group, time of assessment, region of interest and hemisphere. The significance levels for group-by-time interactions effects in individual regions are labelled with stars.

*Region*: Time by group interaction effects were investigated for each region of interest on both hemispheres (Fig. 4). We observed interaction effects in the right caudal middle frontal region [*F*(1,38) = 4.25, *p* ≤ 0.04], in the right pars opercularis [*F*(1,38) = 4.4, *p* ≤ 0.04], in the right pars triangularis [*F*(1,38) = 8.3, *p* ≤ 0.006] and the left pallidum [*F*(1,38) = 4.15, *p* ≤ 0.04] (see regions labelled with stars in Fig. 4, Fig. 7a). However, these interaction effects did not survive Bonferroni correction for multiple comparisons.

#### Results of principal component analyses of changes in the cognitive control networks

Since the repeated measure analyses revealed a three-way group by time by region interaction effect for the right but not the left hemisphere, PCA focused on right-lateralised networks only.

**Fig. 5:**
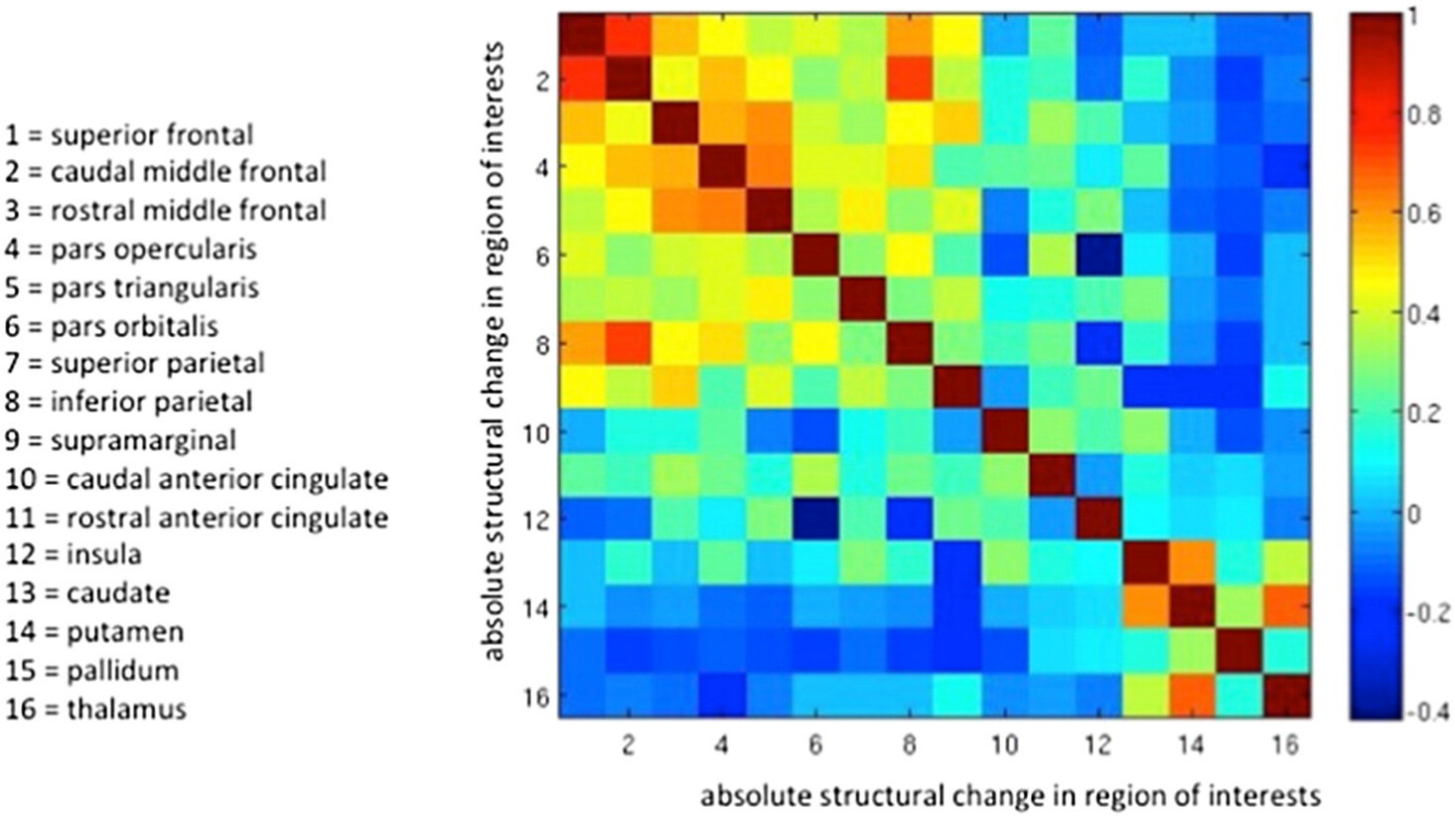
Cross-correlation of structural change across all sixteen regions of interests in the right hemisphere (matrix constructed using Pearson's correlation coefficient).

Fig. 5 shows the results of the Pearson coefficient correlation analyses between changes in cortical thickness and subcortical volume across all sixteen regions of interests of the right hemisphere. Changes in cortical thickness in parieto-frontal regions of the central executive network showed moderate to strong positive correlations with each other, as did changes in subcortical volume within the basal ganglia and thalamus regions. Changes in cortical thickness in the rostral and anterior cingulate correlated positively with each other but only weakly with changes in the insula. Importantly, structural changes in regions of different networks were either not correlated or anti-correlated with each other.

Table 2 summarises the first principal component loadings of changes in cortical thickness and subcortical volume indices for the right-lateralised central executive, anterior salience, and basal ganglia networks respectively. As can be seen six executive brain regions had loadings of > 0.7 and hence contributed most to the summary index of structural changes in the right executive network. These were the caudal middle frontal gyrus, the pars opercularis, pars triangularis, pars orbitalis, as well as inferior and superior parietal cortices.

**Fig. 6:**
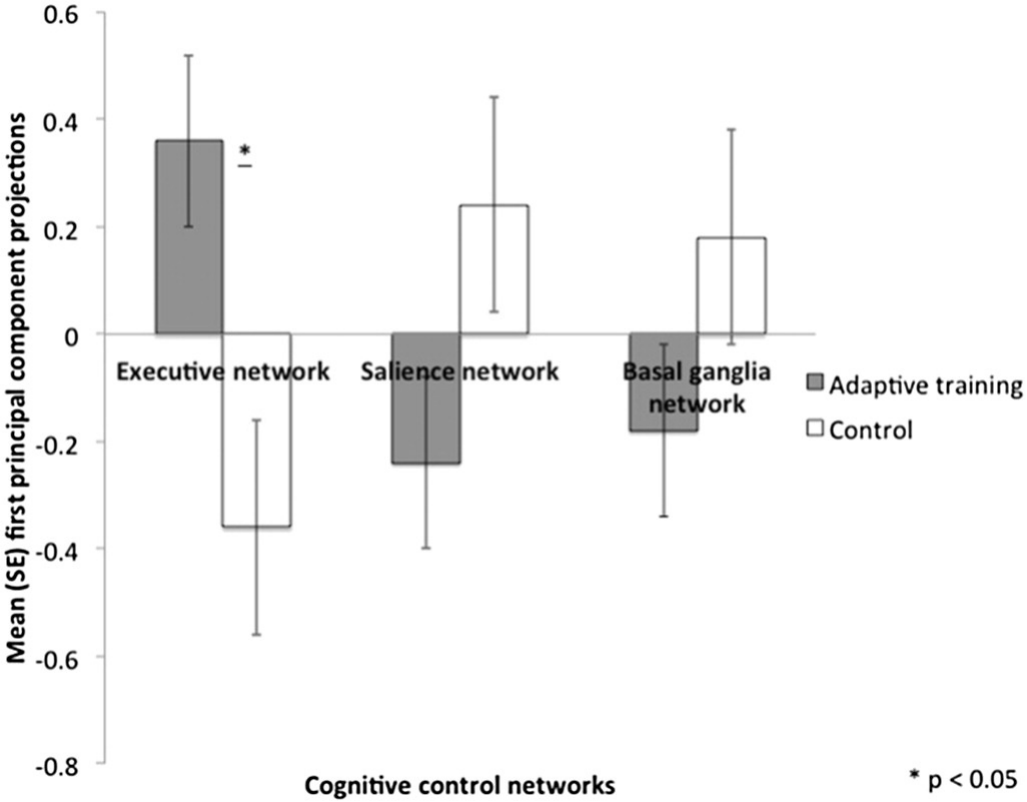
Mean projections on the first principal component as summary indices of structural change across the three cognitive control networks on the right hemisphere for the adaptive training and the control groups. There was a significant group difference (*p* < 0.05) for overall structural changes in the executive network but not for the salience or the basal ganglia network.

Independent *t*-tests on the individual PC projections as summary indices of structural changes across the regions of each network demonstrated significant group differences for the right executive network [*t*(38) = 2.42, *p* = 0.018] but no differences for the salience [*t*(38) = − 1.53, *p* = 0.13] or the basal ganglia networks [*t*(38) = − 1.44, *p* = 0.26] (Fig. 6).

#### Results of logistic regression analysis: Plastic changes predict training group

**Fig. 7:**
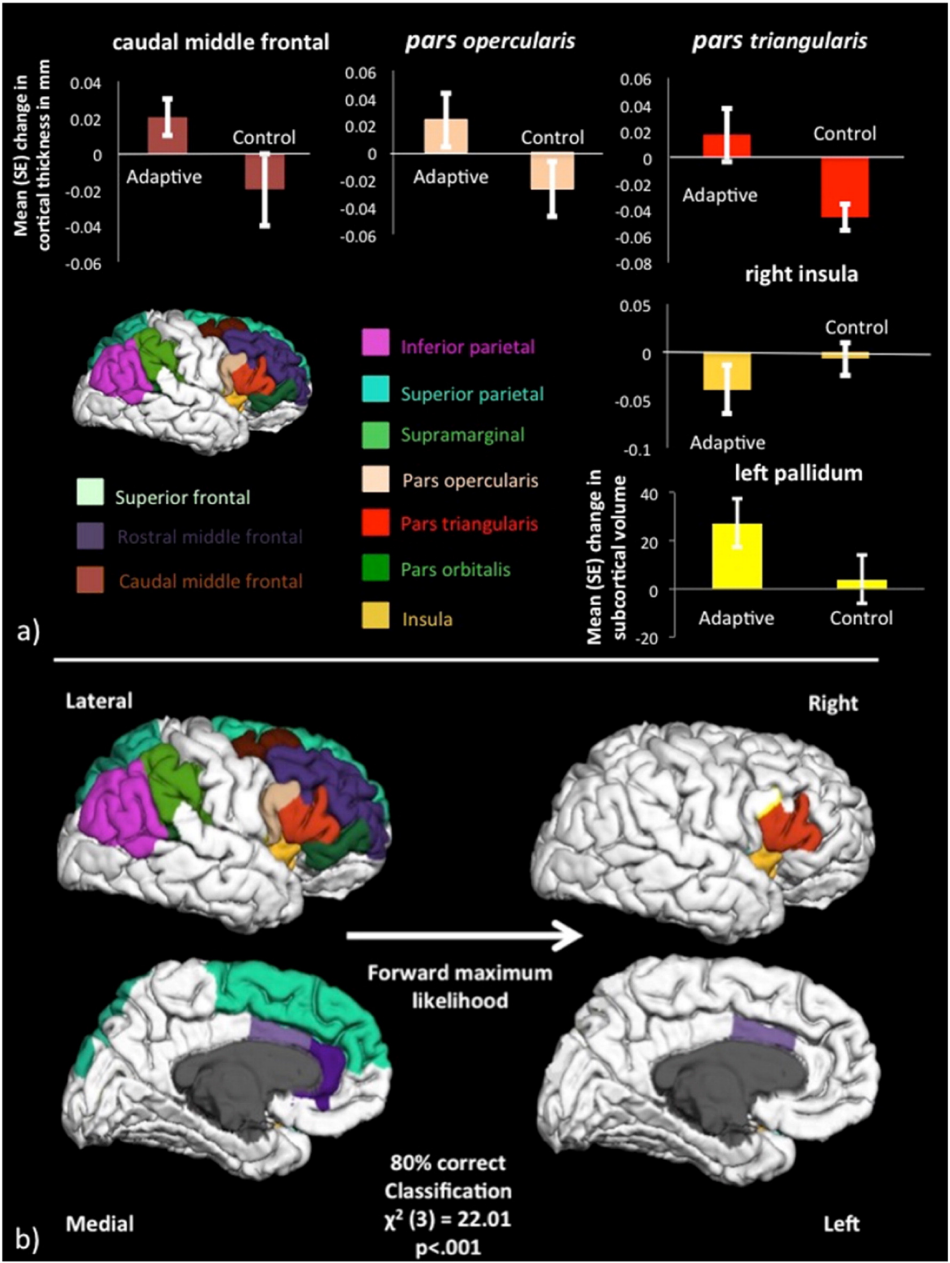
Changes in cortical thickness in response to adaptive working memory training relative to non-adaptive control activities. (a) The top panel displays bar charts of the average changes (SE) in cortical thickness and subcortical volume after adaptive training and control activities for the four regions (right caudal middle frontal, right pars opercularis, right pars triangularis, left pallidum) that showed time by group interaction effects (on the uncorrected level) and the right insula (identified in logistic regression analysis and within-group comparisons). The lateral view on the right cortical surface displays cortical regions of interests selected from Desikan–Killiany atlas (Desikan et al., 2006). (b) The bottom panel displays the results of the logistic regression analyses with training group category (adaptive training versus control) as dependent variable and changes in cortical thickness and sub-cortical volume in the regions of interest as independent variables. A model containing the thickness changes in the left caudal anterior cingulate (light purple), the right *pars triangularis* (red) and the right insula (orange) regions had an overall classification accuracy rate of 80%.

Cortical thickness changes in the left caudal anterior cingulate (Wald *χ*^2^ = 5.2, *p* = 0.02), in the right pars triangularis (Wald *χ*^2^ = 7.8, *p* = 0.005) and the right insula (Wald *χ*^2^ = 6.4, *p* = 0.01) together could identify 85% of the adaptive trainees and 75% of the control participants correctly (Fig. 7b). The overall classification accuracy of the model for the complete dataset (*n* = 40) was 80% [*χ*^2^(3) = 22.01, *p* < 0.001]. The ten-fold cross validation accuracy of classifying novel cases into adaptive training versus control participants correctly was 70%, differing significantly from chance [*χ*^2^(1) = 4.8, *p* = 0.028]. Thus, the type of training condition (adaptive versus control) an individual underwent could be predicted by a unique pattern of brain plasticity across regions of the central executive and anterior salience networks.

Abbreviations: PC = principal component, SE = standard error.

#### Within-group comparisons of directions of change

For those regions identified by the repeated measure ANOVAs (right caudal middle frontal, right pars opercularis, right pars triangularis, left pallidum) and the logistic regression analyses (left anterior cingulate, right insula, right pars triangularis) within-group comparisons between baseline and outcome data were conducted to inform about the direction of structural changes (see Fig. 7a).

Participants in the *adaptive training* group showed a 1% increase in cortical thickness in the right caudal middle frontal gyrus [*t*(19) = − 2.26, *p* = 0.03] [baseline mean thickness = 2.671 mm, SE = 0.026 versus outcome mean thickness = 2.696 mm, SE = 0.023; mean difference = 0.025 mm, SE = 0.01; 95% CI: 0.0052 to 0.046] (*d* = 0.51) and a 2.16% increase in subcortical volume in the left pallidum [*t*(19) = − 2.53, *p* = 0.026] [baseline mean volume = 1261.48 mm^3^, SE = 47.34 versus outcome mean volume = 1288.73 mm^3^, SE = 46.67; mean difference = 27.25 mm, SE = 10.47; 95% CI: 6.54 to 48.15] (*d* = 0.57). They also showed a 1.2% *reduction* in cortical thickness in the right insula [*t*(19) = 2.28, *p* = 0.03] [baseline mean thickness = 3.359 mm, SE = 0.025 versus outcome mean thickness = 3.319 mm, SE = 0.024; mean difference = − 0.04 mm, SE = 0.017, 95% CI: − 0.07 to − 0.007] (*d* = 0.51).

Participants in the *control* group demonstrated a 1.7% *decrease* in thickness in the right pars triangularis [*t*(19) = 4.10, *p* = 0.001] [baseline mean thickness = 2.746 mm, SE = 0.037 versus outcome mean thickness = 2.70 mm, SE = 0.037; mean difference = − 0.045, SE = 0.01, 95% CI: − 0.067 to − 0.024] (*d* = 0.86). No other significant differences were observed (see Table 1 in Metzler-Baddeley et al., 2016, Data in brief).

### Training-induced cognitive changes

Adaptive working memory training improved performance in all trained working memory span tasks whereas performance levels in the control group – *by definition* – did not improve (Table 3 and Fig. 3 left hand panel).

The two groups differed significantly in performance changes in spatial span [*t*(38) = 5.18, *p* < 0.001] and backward digit span [*t*(38) = 4.10, *p* < 0.001] [with a trend for forward digit span, *t*(38) = 2.46, *p* = 0.02] (Fig. 3 right hand panel). Type of training did not affect changes in the remaining cognitive benchmark tests (see Table 2 in Metzler-Baddeley et al., 2016, Data in brief).

### Relationships between training-specific changes in cortical thickness and cognition

**Fig. 8:**
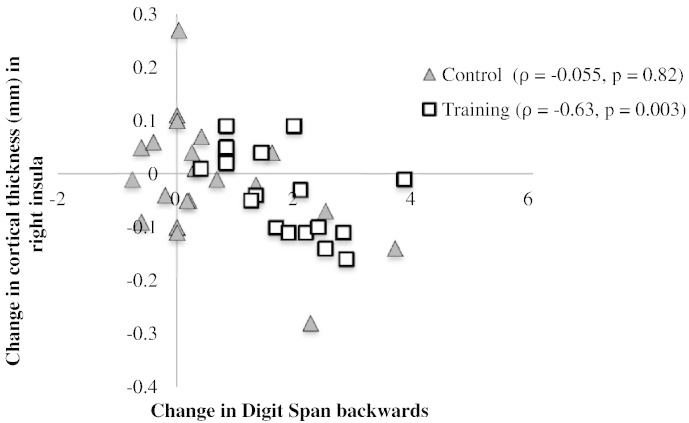
Negative Spearman rho correlation between changes in the thickness of the right insula and performance changes in the backward digit span task for the training group (white squares) compared to no correlation in the control group (grey triangles). The two correlation coefficients differed significantly (*z* = − 1.96, *p* = 0.025). All data were inspected for outliers defined as two standard deviations from the mean score (*n* = 19 in each group).

For the training group performance changes in backward digit span correlated negatively with thickness changes in the right insula (*ρ* = − 0.627, *p* = 0.003, 95% CI: − 0.84 and − 0.31, *n* = 20; with one outlier excluded: *ρ* = − 0.631, *p* = 0.004, 95% CI: − 0.86 and − 0.30, *n* = 19) (Fig. 8). In contrast no correlation was observed for the control group (*ρ* = − 0.189, *p* = 0.43, 95% CI: − 0.59 and 0.26, *n* = 20; with one outlier excluded: *ρ* = −.055, *p* = 0.82, 95% CI: − 0.48 and 0.39, *n* = 19) (Fig. 8). Furthermore the two correlation coefficients from the training and control groups differed significantly from each other, *z* = − 1.96 (*p* = 0.025, 1-tailed). No other significant correlations were present (see Table 3 in Metzler-Baddeley et al., 2016, Data in brief).

### Assessment of test–retest reliability of cortical thickness measures in the left occipital cortex as control region and of intracranial volume

The ICC for the cortical thickness measures obtained at the two time points derived from the left occipital cortex was 0.97 (95% CI: 0.95 to 0.99) demonstrating excellent test–retest reliability. No significant difference between baseline and outcome cortical thickness measures in this region was observed [*t*(39) = 0.66, *p* = 0.51] [baseline mean thickness = 2.447 mm, SE = 0.025 versus outcome mean thickness = 2.441 mm, SE = 0.028; mean difference = − 0.0057, SE = 0.008, 95% CI: − 0.0101 to 0.022]. No evidence of scanner drift effects across time were observed for the occipital region (see Fig. 1 in Metzler-Baddeley et al., 2016, Data in brief).

Furthermore intracranial volume measures were identical between baseline and outcome assessment for the adaptive training (mean ICV = 1,628,696.60 mm^3^; SD = 163,001.34 mm^3^) and for the control group (mean ICV = 1,639,129.90 mm^3^; SD = 108,638.69 mm^3^). However, the control group showed larger ICV at baseline and at outcome (*F*(1, 38) = 4.779, *p* = 0.035).

Positive correlations between the duration of training and performance changes in backward digit span (*r* = 0.54, *p* < 0.001), forward digit span (*r* = 0.38, *p* = 0.016) and spatial span (*r* = 0.37, *p* = 0.018) were present for all participants (*n* = 40) but duration of training did not correlated with structural changes in any of the ROIs (see Table 4 in Metzler-Baddeley et al., 2016, Data in brief).

## Discussion

### Summary of structural results

2 months of adaptive working memory training relative to non-adaptive control activities resulted in a complex pattern of subtle and localised structural changes. We found a four-way interaction effect between group, time, region and hemisphere, which was due to an interaction between time, region and hemisphere for the adaptive but not the control group and due to an interaction effect between group, time and region for the right but not the left hemisphere. Recognising the inter-correlations between structural changes within the proposed cognitive control networks, PCA was employed to create summary indices of structural change for the executive, salience and basal ganglia networks of the right hemisphere. The two groups differed in the PC summary indices in the right-lateralised executive network but not in the right salience or basal ganglia networks. Time by group interaction effects were present at uncorrected significance levels for three right executive regions, i.e., the caudal middle frontal cortex, the right pars opercularis and the right pars triangularis as well as the left pallidum. Together, these results suggest subtle training-related changes in cortical thickness in right-lateralised fronto-parietal executive regions consistent with previous findings of increased BOLD activity, changes in dopamine D1 receptor binding potential, and of grey and white matter indices in these regions (Olesen et al., 2004, McNab et al., 2009, Takeuchi et al., 2010, Takeuchi et al., 2011). Further the observed training-related volume increases in the left pallidum are consistent with previous reports of BOLD activity changes in the left striatum after working memory training (Dahlin et al., 2008, Kühn et al., 2013). It is also worth mentioning here that we observed relatively large group differences in subcortical volume at baseline and outcome in the caudate and the putamen that might potentially have masked any differential group effects across time in these regions. Nevertheless the observed training-related changes in the left pallidum provide further evidence for an important role of the basal ganglia system in mediating working memory function.

We conducted a complementary logistic regression analysis to answer the question of whether it was possible to predict the type of training participants had undergone from the observed pattern of structural changes. This analysis identified changes in the right *pars triangularis*, the right insula and the left anterior cingulate as best predictors for group allocation, suggesting that not only changes in executive but also in salience network regions distinguished between the two groups.

Since the omnibus analyses alone did not inform about the *direction* of changes, we further carried out exploratory within-group comparisons between baseline and outcome data in the identified regions. These analyses revealed for the adaptive group a 1% *increase* of cortical thickness for the right caudal middle frontal region, a 2.2% *increase* of subcortical volume in the left pallidum and a 1.2% *reduction* of cortical thickness in the right insula. Furthermore and unexpectedly, there was a 1.7% *reduction* of thickness in the right pars triangularis for the control group.

### Summary of cognitive results

Adaptive training resulted in robust performance improvements in all trained Cogmed tasks as well as in the near transfer working memory tasks of backward digit span and spatial span (with a trend for forward digit span). However, no transfer effects of training on cognition in other benchmark executive function tasks were observed. This lack of transfer to other cognitive domains is consistent with accumulating evidence suggesting that transfer effects are rarely detected (Jaeggi et al., 2014, Owen et al., 2010, Redick et al., 2013) and that generalisation may only occur when cognitive domains and supporting neural networks overlap sufficiently between training and benchmark activities (McNab and Klingberg, 2008). Recent meta-analyses of transfer effects of working memory (Au et al., 2015) concluded that generalisation effects may occur in healthy adults but that effect sizes are small and large sample sizes seem to be required to detect them (Corbett et al., 2015). However, this position has been questioned by Melby-Lervag and Hulme (2015) who found no evidence for transfer effects of working memory training after restricting their meta-analyses to rigorously controlled studies (including active control groups) and after taking baseline differences between groups into account in their effect size calculation. Consistent with Melby-Lervag et al., our results suggest that working memory training does not lead to transfer effects in healthy adults, although it remains possible that such effects may be detectable in considerably larger sample sizes (Corbett et al., 2015).

### Relationship between structural and cognitive changes

Structural changes in the right executive regions and the left pallidum did not correlated with improvements in working memory span performance (see Table 3 in Metzler-Baddeley et al., 2016, Data in brief) but instead thickness changes in the right insula were negatively correlated with improvements in backward digit span performance (Fig. 8) reflecting that participants in the adaptive group with the largest thickness reductions also exhibited the largest training related behavioural improvements. This pattern of results was only observed for the training but not the control group suggesting that plastic changes in the salience network appeared to have contributed to the behavioural improvements after working memory training. Since we did not observe any cognitive transfer effects to other tasks it was not possible to assess whether changes in the pallidum were related to any generalisation effects.

### Interpretation of training-related changes structural changes

Group by time interaction effects are considered the gold standard evidence for training-specific plastic and behavioural changes (Thomas and Baker, 2013). Significant interaction effects are thought to reflect intervention-specific changes across time in the absence of changes in control conditions. Inspection of Fig. 4, Fig. 7, however, reveal that in the present study we observed group by time interaction effects in ROIs that exhibited opposing effects in both groups. From Fig. 4 we can see that cortical thickness measures in the adaptive group showed small increases in right-lateralised fronto-parietal regions and small reductions in anterior salience regions whilst the control group seemed to exhibit subtle reductions in almost all cortical regions on the right hemisphere. Indeed from Fig. 7 it becomes clear that the group by time interaction effect for the right pars triangularis was primarily driven by the thickness reductions in the control group. We found significant group by region interactions for both baseline and outcome data and from Fig. 4 it can be seen that the control group appears to have larger baseline thickness in the right pars triangularis than the adaptive group, which may have contributed to the relatively larger thickness reduction for control participants in this region. However, this descriptive baseline difference (Control: mean = 2.7462, SD = 0.168 versus training: mean = 2.6659, SD = 0.136) was not significant [*t*(38) = − 1.656, *p* = 0.106]. In addition, on the descriptive level, small reductions in thickness after control activities seemed apparent in the majority of right-lateralised cortical regions including in ROIs without baseline differences (e.g. see right caudal middle frontal cortex in Fig. 4). Reductions in cortical thickness were also observed for the right insula after adaptive training and descriptive baseline difference may have contributed (see Fig. 4) (Control: mean = 3.31, SD = 0.11 versus training: mean = 3.35, SD = 0.11) but again were not found to be significant [*t*(38) = − 1.58, *p* = 0.12]. It should be noted that no group by time interaction effect was evident for the right insula (see Fig. 4, Fig. 7) but that this region was identified by the logistic regression and follow-up within-group comparisons. Importantly, reductions in the right insula after adaptive training were correlated with working memory span improvements.

On balance, we cannot completely rule out that baseline differences may have contributed to the observed reductions in thickness, but we also need to consider the possibilities that i. adaptive training might have resulted in thickness reductions in the right insula and ii. control activities might have resulted in reductions of cortical thickness in the right pars triangularis.

There are some reports of training or experience-related opposing structural changes in the literature. Although the majority of structural plasticity studies reported training induced *increases* in grey matter volume or cortical thickness in task relevant regions (Valkanova et al., 2013) there are also a number of reports of *reductions* in grey matter volume or thickness after learning or experience. One of the first plasticity studies found both larger grey matter volumes in posterior and smaller volumes in anterior hippocampal regions in experienced taxi drivers compared to controls (Maguire et al., 2000). Individual differences in volume in the posterior hippocampus correlated positively with experience as taxi driver whilst volume differences in the anterior hippocampus were negatively related to experience. Further, Taubert et al. (2010) reported time and brain location dependent increases and reductions in grey matter volume associated with balancing training and Takeuchi et al. (2011) found reductions of grey matter volume in parieto-frontal regions after an intensive working memory training of 4 h per day for 5 days. Together these results indicate that the direction of observed plastic changes may be dependent on the brain location as well as the time schedule and duration of training.

Accumulating neuroimaging evidence suggests that executive and salience networks may be functionally dissociable (Elton and Gao, 2014, Menon and Uddin, 2010). Recently, the effects of adaptive working memory training were compared with effects of meditation training, such as relaxation or mindfulness, across executive and salience networks (Tang and Posner, 2014). In contrast to working memory training, advanced meditation training led to reductions in parieto-frontal BOLD activity and to increases in activity in anterior cingulate and insula regions. Here, we observed a complementary pattern of increased cortical thickness in the middle frontal gyrus paired with reductions in thickness in the right insula after working memory training. Bearing in mind the caveat of directly comparing functional and structural MRI evidence, one may speculate that adaptive training up-regulates recruitment of parieto-frontal regions to focus attention on demanding tasks whilst concurrently down-regulating recruitment of salience regions which are not essential for efficient task performance. The detection of potentially distracting salient information may even hamper successful task completion. Such a pattern of opposing structural changes might reflect highly adaptive and dynamic processes to regulate neuronal activity across the brain with the aim to successfully complete a task by allocating resources to brain areas necessary for task completion whilst minimising energy consumption in task irrelevant regions (Laughlin and Sejnowski, 2003). Although speculative, the consequence of such processes could be that exercising a particular function may achieve specialisation of the brain in performing this task to the potential detriment of other functions.

The principle of energy conservation in the brain may also provide a potential explanation for our unexpected finding of *reductions* of cortical thickness the right pars triangularis, after undemanding control activities. The right pars triangularis forms the mid-portion of the ventro-lateral prefrontal cortex and is part of the right lateralised fronto-parietal attention network (Aron et al., 2004, Corbetta et al., 2008). In fMRI studies this region appeared uniquely activated in situations of response uncertainty where participants engage in minimal or no preparation prior to the occurrence of a stimulus (Levy and Wagner, 2011). Thus, reductions in thickness in this region may reflect the fact that participants were performing unchallenging tasks on “autopilot” without having to allocate attention resources to task preparation and execution. Anecdotal feedback from some participants in the control group of “switching off” or “meditating” whilst performing the training together with Tang et al.'s findings of reduced fMRI activity in parieto-frontal regions after meditation training would be consistent with such a disengagement interpretation. Disengaging or down-regulation of signalling in executive regions that is not essential for the completion of low demanding tasks would allow the brain to minimise resources required for task completion and hence conserve energy (Laughlin and Sejnowski, 2003). It should be noted that based on the preliminary evidence from the current study these interpretations remain highly speculative. Future research is required to answer the question if high compared to low demanding activities lead to opposing structural changes compared to a passive control group without intervention.

### Biological interpretation of changes in cortical thickness

The cellular basis of grey matter changes measured with MRI remains poorly understood (Kanai and Rees, 2011) and requires further investigation to clarify whether any changes in the microstructural environment that alter T_1_ locally may lead to *apparent* macrostructural changes in thickness. Increases in grey matter volume have been interpreted in terms of training-induced neurogenesis or growth of glia cells and/or synapses (Zatorre et al., 2012). Adult neurogenesis in the subventricular zone of the lateral ventricles and the hippocampus is well established (Lacar et al., 2014). However, evidence for neurogenesis in the mammalian neocortex remains weak (Au and Fishell, 2006). Learning-induced glia- or synaptogenesis are potentially more likely candidates that may underpin structural MRI changes (Dong and Greenough, 2004, Kleim et al., 1996, Kleim et al., 2007). Selective pruning or elimination of redundant axons, dendrites or synapses may also facilitate brain plasticity (Yasuda et al., 2011). For example, Knafo et al. (2005) demonstrated that olfactory-discrimination learning in rats was associated with both strengthening of spine densities for reinforced and pruning of spine densities for weakened associations. It seems likely that brain plasticity draws on several neural and non-neural mechanisms of learning and that these mechanisms operate on different time scales. Thus, structural changes observed with MRI may reflect a combination of all contributing mechanisms at a given time point. Hence, although thickness increases are often interpreted in terms of glio- or synaptogenesis and reductions in terms of selective pruning and elimination, these inferences may be insufficient if plastic mechanisms operate on different time scales (Kanai and Rees, 2011, Zatorre et al., 2012). The present study did not acquire any MRI data during the training phase and hence was not designed to detect potential differences in the time course of plastic changes. Future studies may include multiple time points of assessment to help disentangling the underlying processes that result in measurable changes in grey matter thickness or volume measures.

### Potential artefacts in the MRI cortical thickness measures and the experimental design

Estimates of cortical thickness were derived with the longitudinal FreeSurfer analysis stream from T_1_-weighted MRI images and interpolated for subdivisions of the cortex following the Desikan–Killiany atlas. Precise estimates of cortical thickness for different regions of interests require that the boundary between grey and white matter must be accurately delineated on these T_1_-weighted images. The FreeSurfer methods were chosen for their excellent reliability (Desikan et al., 2006, Dickerson et al., 2008, Reuter et al., 2012) and demonstrated sensitivity to small changes (Sabuncu et al., 2014). In this study test–retest reliability of cortical thickness measures derived from left occipital cortex were highly precise (ICC = 0.97) and no differences in intracranial volume between the two time points were present in either groups. Further the chosen FreeSurfer interpolation methods had the advantage that cortical thickness maps were not restricted to the voxel resolution of the original data and hence it was possible to detect sub-millimeter differences within the range of 0.025 mm to 0.045 mm. These *apparent* changes in cortical thickness were consistent with effect sizes of training reported in previous studies (Engvig et al., 2010).

Nevertheless systematic biases in cortical thickness indices could potentially have occurred for instance due to group differences in movement artefacts, in time-of-day scanning effects or scanner drift effects which could have led to incorrect segmentation of grey and white matter boundaries. All data were visually quality inspected by an operator (KC) blind to the learning condition and no systematic differences in terms of movement artefacts were detected between the groups.

To account for MRI scanner related artefacts such as drift effects and time-of-day scanning biases MRI sessions for participants from both groups were interleaved. Subsequent analyses did not detect a systematic bias of time-of-day of scanning nor did we observe any scanner drift effects in cortical thickness data derived from the occipital lobe (see Fig. 1 in Metzler-Baddeley et al., 2016, data in brief). There were also no changes in cortical thickness in the occipital control regions across time for either groups and we observed opposing changes in thickness across different ROIs for the training group making a systematic over- or underestimation of thickness measures unlikely.

For the present study we recruited psychology-naive participants who were pseudo-randomly allocated to either the training or the control group ensuring both groups were comparable with regards to demographic and baseline cognitive variables. All participants were blind to their training conditions and at debriefing the majority of participants had not realised the specific training manipulations. The two groups were matched as closely as possible with regards to the learning environment i.e., all participants trained at home and were exposed to the same computerised training environment and the same training tasks. Both groups were also monitored equally and received the same number of feedback emails. The number of training sessions was identical for the groups but the time spent actively on training differed between the groups due to the nature of shorter span control activities. Duration of training was not related to changes in cortical thickness suggesting that time spent exercising per se was not critical for the structural changes. However duration of training was positively related to improvements in working memory span tasks and were likely based both on task complexity and on the time spent exercising. Even in a well-controlled design it cannot be ruled out that potential confounding factors such as differences in training induced expectations, perceptions, motivations or prior knowledge may have contributed to some of observed plastic changes (Boot et al., 2011). Indeed our explanation for the reduction in cortical thickness in the pars triangularis region observed for the control group acknowledges the possibility that motivational factors or attention disengagement may have contributed to this effect.

Despite our efforts of matching both training groups as closely as possible in terms of demographics and baseline cognitive performance we observed a number of baseline structural differences between the groups which may have impacted on the observed changes across time. Inter-individual differences in brain structure are well documented (Braver et al., 2010) and need to be taken into account when interpreting longitudinal changes in brain structure. However it is difficult to see how baseline differences can be avoided. One could pre-scan participants prior to group allocation to measure brain structure in regions of interest but it seems unfeasible to match participants for structural measurements in all brain regions and such a procedure would be very expensive and time consuming.

It should also be noted that although our sample sizes of 20 participants in each group were in keeping with previous training studies, there were a number of trends in our data, which did not reach significance after multiple comparison corrected level of significance.

### Summary and conclusion

In a robustly designed study we demonstrated that 2 months of adaptive working memory training compared to non-adaptive control activities resulted in subtle plastic changes that varied in direction dependent on the brain location and training complexity. The observed effects were small but consistent across participants. The observations of localised and subtle training effects are consistent with previous reports and highlight the importance for carefully designed and hypotheses driven studies to ensure sufficient power. Our findings demonstrate that highly taxing working memory training can induce structural alterations in the right-lateralised central executive network and left pallidum with some preliminary evidence for thickness reductions in the anterior salience network which were correlated with training-induced improvements in working memory span capacity. Further we observed some evidence for changes in cortical thickness after control training suggesting the possibilities that repetitive and undemanding activities may be associated with reductions of thickness in the brain. Together the observed pattern of results demonstrates that activity-related plastic changes depend in their direction on the brain location and task complexity, consistent with the view that efficient processing in the brain is governed by principles of energy conservation.

## Figures and Tables

**Table 1:** Summary of demographic variables and mean (standard deviation) performance in working memory and executive function benchmark tests of the two groups at baseline and results of independent *t*-tests.

	Training	Controls	*t*_(38)_	*p*
*n*	20	20	–	–
Age (years)	26 (6.2)	27 (6.8)	0.44	0.67
Females	11	10	–	–
Right-handed	19	20	–	–
Forward digit span	5.3 (0.8)	5.2 (0.7)	0.67	0.51
Backward digit span	4 (1.4)	4 (1.4)	0.01	0.99
Spatial span	5 (0.5)	4.9 (0.5)	0.97	0.34
Double trouble	22.8 (13.6)	25.9 (15.4)	0.69	0.49
Grammatical reasoning	0.79 (0.2)	0.73 (0.2)	0.97	0.34
Tree task	23.7 (8.7)	19.8 (7.2)	0.15	0.93
Odd one out	9.5 (3.2)	9.1 (4.3)	0.37	0.71
Self-ordered search	6.2 (1.1)	5.5 (1.4)	0.18	0.07
Complex span	25.3 (6.5)	22.6 (7.9)	0.12	0.25
Number of training sessions	40	40	–	–
Active time per session (min)	42.7 (4.65)	36.31 (6.15)	3.75	0.001
Average time of day (h:min) of baseline MRI scan (range)	13:06 (9:37–18:17)	13:44 (9:47–18:31)	0.16	0.88
Average time of day (h:min) of outcome MRI scan (range)	13:14 (9:03–17:29)	14:31 (10:04–18:16)	1.7	0.09

**Table 2:** First principal component loadings of changes in cortical thickness across regions of the right-lateralised central executive, anterior salience and basal ganglia networks. Principal component analyses were conducted across all participants (*n* = 40) for regions of each network separately. Principal component loadings > 0.7 are highlighted in bold.

First principal component of change in cortical thickness	
*Right central executive network*	
Caudal middle frontal	**0.78**
Rostral middle frontal	0.60
Superior frontal	0.59
Pars triangularis	**0.72**
Pars opercularis	**0.78**
Pars orbitalis	**0.77**
Superior parietal	**0.73**
Inferior parietal	**0.78**
Supra marginal	0.59

*Right anterior salience network*	
Caudal anterior cingulate	**0.84**
Rostral anterior cingulate	0.66
Insula	0.45

*Right basal ganglia network*	
Caudate	**0.75**
Putamen	**0.93**
Pallidus	0.43
Thalamus	**0.79**

**Table 3:** Results of the within group comparisons between performance in all eleven Cogmed tasks after the first and last training sessions for the adaptive training group (*n* = 20).

	*t*(19)-statistic	*p*-value
3D cube	15.59	< 0.001
Rotating data link	21.33	< 0.001
Input module	12.24	< 0.001
Input module with lid	12.13	< 0.001
Visual data link	21.23	< 0.001
Space whack	13.15	< 0.001
Asteroids	23.12	< 0.001
Data room	14.63	< 0.001
Decoder	3.02	= 0.007
Sorter	16.9	< 0.001
Rotating dots	15.64	< 0.001

## Footnotes

1 Cogmed and Cogmed Working Memory Training are trademarks, in the US and/or other countries, of Pearson Education, Inc. or its affiliate(s).
